# Concurrent and Predictive Associations between the Life's Simple 7 & Brain Structure in Middle-Age and Early-Old Age

**DOI:** 10.1093/geroni/igab046.3573

**Published:** 2021-12-17

**Authors:** Teresa Warren, McKenna Williams, Christine Fennema-Notestine, Jeremy Elman, Jennifer de Anda, William Kremen, Carol Franz

**Affiliations:** 1 San Diego State University, San Diego, California, United States; 2 University of California San Diego, La Jolla, California, United States; 3 San Diego State University, La Jolla, California, United States

## Abstract

American Heart Association’s (AHA) Life’s Simple 7 (LS7), an index of cardiovascular health risks, has been associated with worse brain outcomes but few examined this relationship in midlife. We examined whether LS7 scores at midlife were associated with brain morphometry in early old age. Participants were 471 men who participated in the Vietnam Era Twin Study of Aging. The LS7 index was assessed at mean age 62 (range 55-66) and 68 (range 61-71) and included smoking, physical activity, diet, body mass index, cholesterol, glucose, and blood pressure. Each factor was coded, per AHA criteria, on a 3-point scale (0/poor-2/ideal) and summed to create a composite score (0-14). At mean age 68, participants underwent structural magnetic resonance imaging, which was used to create the previously validated brain measures. Scores included: the ratio of abnormal white matter to white matter, and two Alzheimer’s disease brain signatures (cortical thickness/volume signature and a mean diffusivity (MD) signature). Analyses controlled for age, education, income, ethnicity, and APOE genotype. Concurrently at mean age 68, the LS7 was associated with cortical thickness/volume (F=4.85, p = .028), MD (F=10.89, p = .001) signatures and abnormal white matter ratio (F=14.04, p < .001). Prospectively, the LS7 at mean 62 was significantly associated with age 68 cortical thickness/volume (F=5.08, p = .025) and MD (F=5.54, p = .019) signatures but not with abnormal white matter ratio. These results suggest that prevention strategies that promote heart healthy behaviors could have implications for healthy brain aging.

